# A Geriatric Case of Tourniquet-Like Foot Scarring Caused by Loose Woolen Stockings Successfully Treated at Home

**DOI:** 10.7759/cureus.99739

**Published:** 2025-12-20

**Authors:** Shoryu Takayama, Kosuke Noguchi, Masaya Taniguchi, Asako Watanabe

**Affiliations:** 1 Surgery, Yoshida Clinic Meisei, Aichi, JPN; 2 Surgery, Yoshida Clinic Meisei, Nagoya, JPN; 3 Cardiology, Yoshida Clinic Meisei, Nagoya, JPN; 4 Palliative Care, Yoshida Clinic Meisei, Nagoya, JPN

**Keywords:** elderly patient, foot necrosis, home medical care, tourniquet syndrome, wound care management

## Abstract

A 92-year-old woman developed severe edema and subcutaneous hemorrhage on the dorsal aspect of her right foot, likely due to chronic venous and lymphatic congestion induced by loose woolen stockings. The condition progressed to necrotic skin changes with infection. Through stepwise drainage, debridement, and appropriate topical therapy, complete epithelialization was achieved without hospitalization. This case highlights the importance of early recognition of mild compression-induced circulatory disorders and demonstrates that even severe lower limb ulcers in frail elderly patients can be successfully managed under home medical care with proper wound management and teamwork.

## Introduction

Tourniquet syndrome refers to a constrictive injury resulting from mechanical compression that impedes venous and lymphatic return, eventually leading to ischemia and tissue necrosis [1]. Although it is most commonly described in pediatric cases caused by hair or threads wrapping around digits, similar pathophysiology can occur in adults and the elderly when external compression, such as from elastic bands, stockings, or clothing, induces localized circulatory impairment [2,3]. In frail older adults with chronic edema, impaired mobility, and fragile skin, even mild or unintentional compression may cause subcutaneous hemorrhage, ischemia-reperfusion injury, and subsequent infection [4]. Several reports have described compression-induced ulcers or necrosis caused by medical devices or garments; however, such injuries are often underrecognized in community or home-care settings [5]. Such injuries may progress insidiously due to reduced pain perception and self-care capacity in elderly individuals. Furthermore, once ulceration occurs, the healing process is often prolonged because of decreased vascular reserve, malnutrition, and impaired wound healing capacity [6]. In Japan and other countries with aging populations, home-based medical care is increasingly important for managing chronic wounds [7,8]. However, few case reports have documented successful home management of severe necrotic wounds induced by compression mechanisms [9]. This case highlights a rare instance of tourniquet-like foot necrosis caused by loose woolen stockings in a non-hospitalized elderly patient, which was successfully treated through stepwise drainage, debridement, and wound care under home medical supervision. The case underscores the importance of early recognition of compression-induced circulatory disorders and demonstrates the feasibility of comprehensive wound management in the home-care environment.

## Case presentation

**Figure 1:**
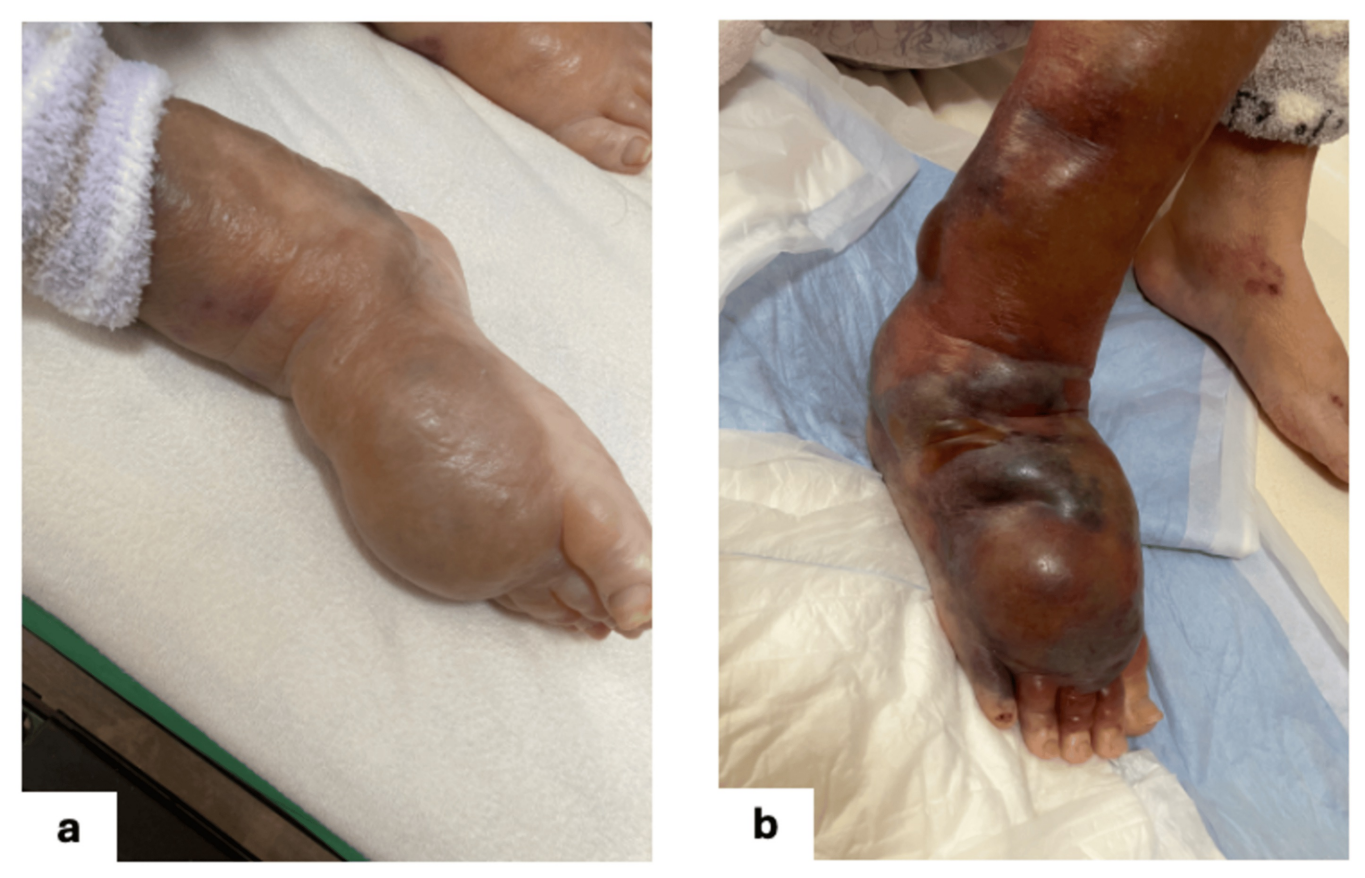
Development of compression-induced edema and subcutaneous hematoma prior to necrosis The patient's skin was fragile, and woolen stockings were used for protection. This led to the development of subcutaneous edema (1a). The patient fell, resulting in a hematoma forming within the subcutaneous edema (1b).

A 92-year-old woman with a medical history of rheumatoid arthritis, reflux esophagitis, hypertension, and immune thrombocytopenia (ITP) presented with severe swelling and subcutaneous hemorrhage on the dorsum of her right foot. Her regular medications included amlodipine, prednisolone (5mg, for about 30 years), and esomeprazole. She had been wearing loose woolen stockings for skin protection, which had gradually dug into the skin and caused subcutaneous edema (Figure 1a). Subsequently, she fell, resulting in a hematoma developing within the edematous tissue (Figure 1b).

**Figure 2:**
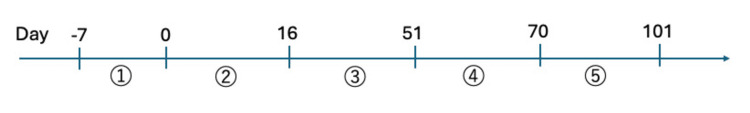
Clinical time course ① Before onset : The wool stockings worn for skin protection had dug slightly into the skin. This caused significant subcutaneous edema. The patient fell on Day 0, resulting in a hematoma within the subcutaneous edema. ② Initial treatment phase : As initial management, the hematoma was incised to drain fluid, and a drain was placed. The subcutaneous tunnel formation site was laid open. For infection control, the patient was prescribed oral cefcapene pivoxil for 3 days. ③ Maintenance phase : Due to the patient's predisposition to edema, significant exudate was present. Cadexomer iodine ointment was used for infection control and to remove excess fluid from the wound site. ④ Additional surgery + preservation treatment period : A subcutaneous tunnel resistant to conservative treatment developed, necessitating lay-open. As exudate decreased, treatment was switched to prostaglandin ointment for conservative management. ⑤ Epidermal formation period : Once epidermal formation was achieved for the first time, moisturization with Propet ointment was initiated, and treatment was deemed complete.

The chronological progression and treatment process are summarized in Figure 2.

Initial presentation

**Figure 3:**
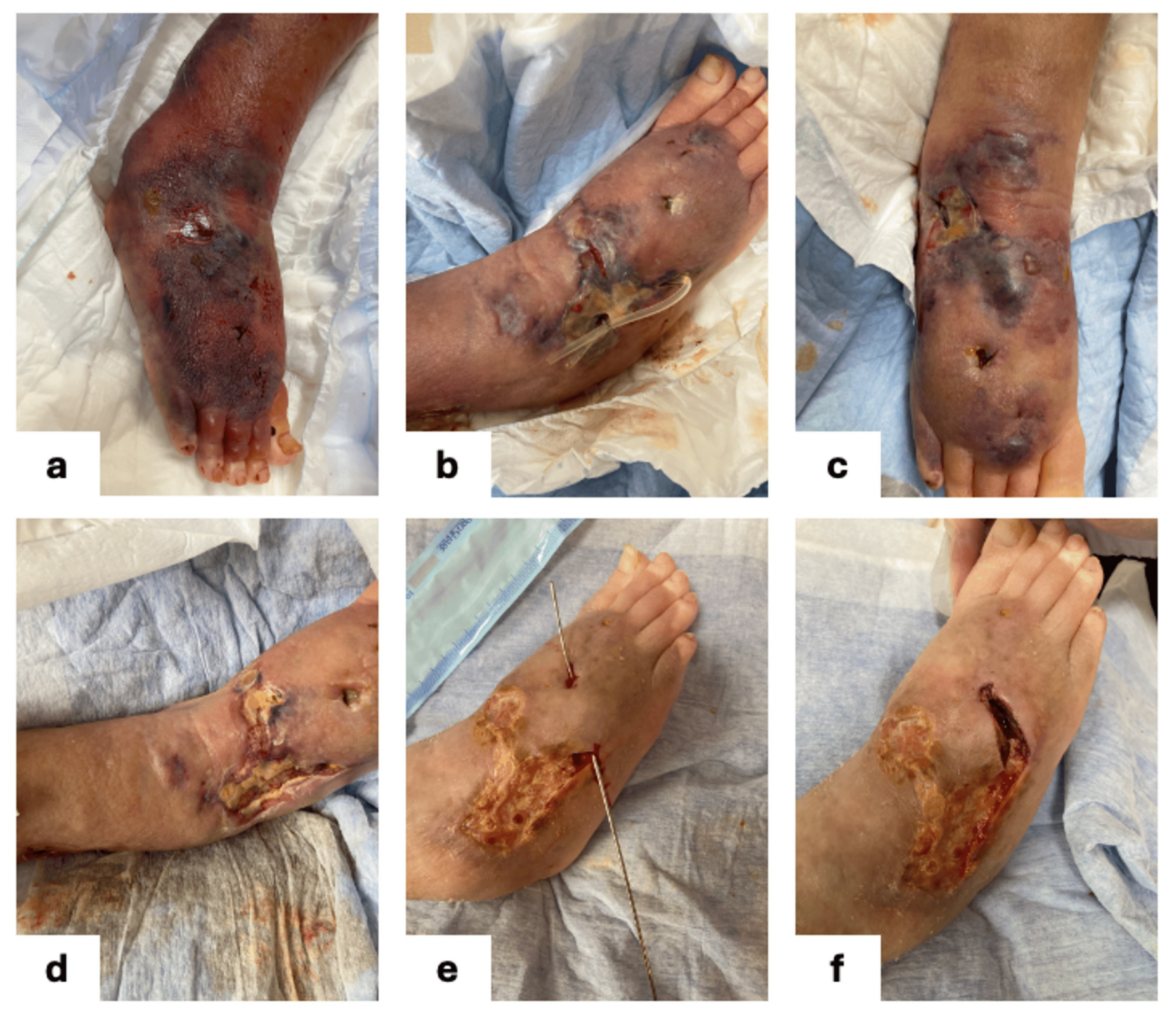
Treatment procedure The hematoma was incised and drained (3a). A subcutaneous tunnel was identified and treated with a drain, followed by a lay-open (3b,3c). The wound site had significant exudate and was treated with Cadexomer iodine ointment (3d). A subcutaneous tunnel appeared and was lay-open (3e,3f).

At the first evaluation, tense edema and a large subcutaneous hematoma were observed. The hematoma was incised and drained, and a Nelaton catheter was placed to maintain drainage (Figure 3a). The surgery was performed in a home setting without adherence to sterile technique. Local anesthesia was administered using 1% Xylocaine. A subcutaneous tunnel was identified, and a lay-open procedure was done to ensure continuous drainage (Figure 3b-3c). The patient was prescribed oral cefcapene pivoxil for three days for the prevention of infection.

Therapeutic maintenance phase

Due to the patient’s predisposition to edema, significant exudate persisted. Cadexomer iodine ointment was used to reduce exudate and maintain infection control (Figure 3d). 

Additional surgery and transition to conservative care

A new subcutaneous tunnel resistant to conservative therapy developed and was subsequently laid open (Figure 3e-3f). The surgery was performed in a home setting without adherence to sterile technique. Local anesthesia was administered using 1% Xylocaine. As the amount of exudate decreased, topical treatment was switched to prostaglandin ointment to promote granulation and epithelialization.

Epidermal formation and recovery

**Figure 4:**
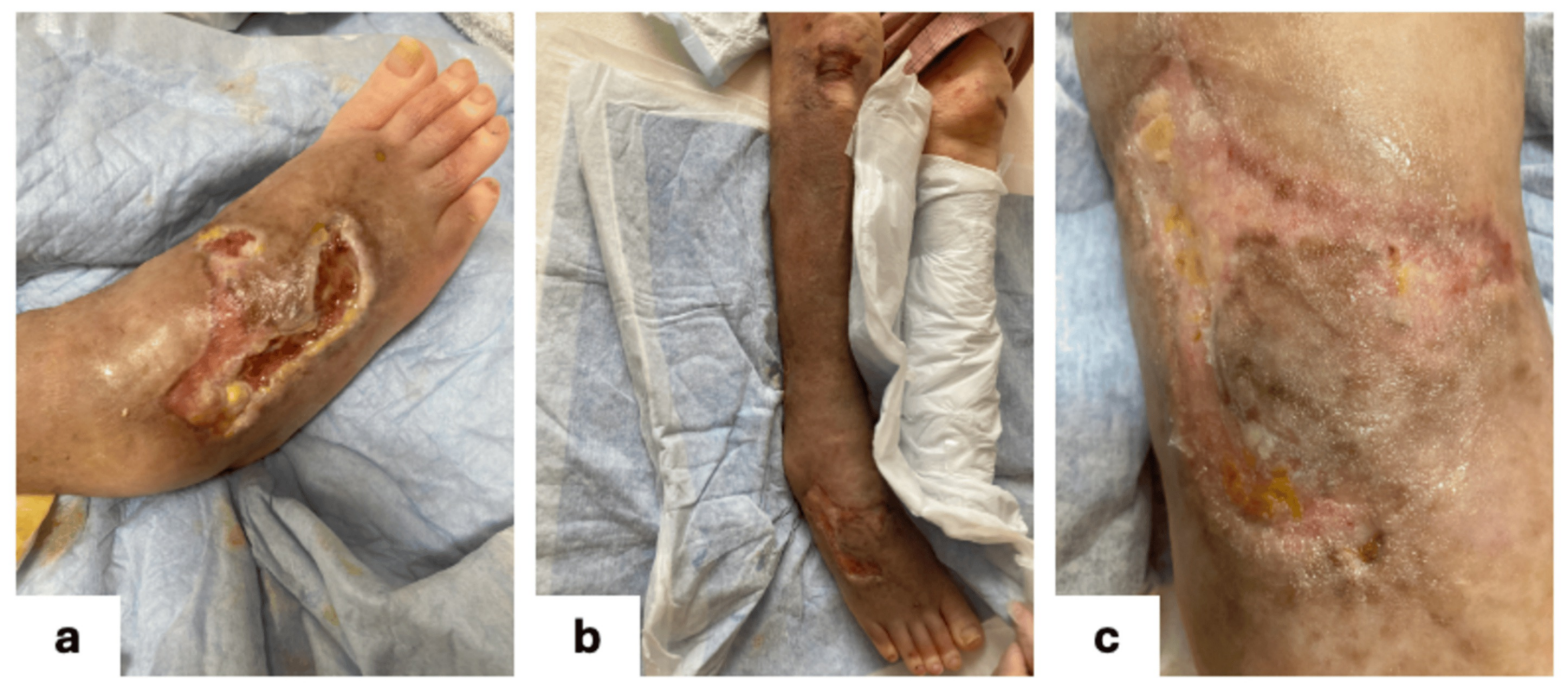
Epidermal formation period Exudate decreased, and treatment was switched to prostaglandin ointment (4a). Since then, epithelialization has progressed, and no subcutaneous edema has developed in the lower limbs (4b,4c).

Once epithelialization was first observed, moisturizing therapy with white petrolatum was introduced (Figure 4a). Progressive epithelial closure and resolution of lower-limb edema followed (Figure 4b, 4c). Complete epithelialization was achieved without hospitalization. No systemic infection occurred, and no intravenous antibiotics were required. All debridement, drainage, and wound-care procedures were conducted entirely in the patient’s home under physician and visiting-nurse supervision.

**Table 1:** Temporal changes in laboratory data during the clinical course Laboratory values are presented from two clinical time points: the initial treatment phase and the maintenance phase. WBC: white blood cell count; RBC: red blood cell count; Hb: hemoglobin; Hct:= hematocrit; Plt: platelet count; γ-GTP: gamma-glutamyl transpeptidase; BUN: blood urea nitrogen; AST: aspartate transaminase; ALT: Alanine aminotransferase

Test	Initial treatment phase	Maintenance phase
Albumin (g/dL)	3.1	3.2
Direct Bilirubin (mg/dL)	0.1	0.1
Indirect Bilirubin (mg/dL)	0.3	0.2
AST (U/L)	27	20
ALT (U/L)	46	31
γ-GTP (U/L)	56	55
BUN (mg/dL)	14.8	12.6
Creatinine (mg/dL)	0.65	0.66
Sodium (mEq/L)	144	142
Potassium (mEq/L)	3.8	3.9
Chloride (mEq/L)	108	105
Blood Glucose (mg/dL)	157	116
CRP quantitative (mg/dL)	0.1	0.1
WBC (×10^3^ / µL)	10.7	8.9
RBC (×10^6^ / µL)	3.39	3.37
Hb (g/dL)	10.1	10.1
Hct (%)	32.6	32.2
Plt (×10^3^ / µL)	118	109

## Discussion

This case illustrates that chronic, low-grade compression can induce tourniquet-like injury resulting in subcutaneous hematoma, necrosis, and delayed healing in frail elderly patients. The underlying mechanism likely involves venous and lymphatic stasis, ischemia-reperfusion injury, and secondary infection [3,4]. Long-term corticosteroid use may also have contributed to skin fragility and impaired wound healing [5]. Previous literature primarily describes tourniquet syndrome in pediatric or accidental contexts, but few reports focus on elderly patients or stockings as the cause [9]. In this case, despite extensive tissue damage, infection control and wound healing were achieved without hospitalization. The outcome underscores the potential for successful home-based wound care when surgical principles-adequate drainage, debridement, and moisture balance-are properly implemented [5]. This report also highlights the critical role of interdisciplinary collaboration between physicians and home-visiting nurses. Early identification of circulatory impairment, consistent wound evaluation, and appropriate topical therapy (e.g., cadexomer and prostaglandin ointments) were essential to recovery. As the global population ages, similar cases may become more frequent, and clinicians should be aware that even mild compression from garments or assistive devices can result in severe skin necrosis in frail elderly individuals. Finally, this case emphasizes that comprehensive wound management, including decompression, drainage, and meticulous follow-up, can be safely performed in the home medical care setting. The successful recovery achieved here without intravenous antibiotics or hospitalization suggests a feasible model for managing complex wounds in aging societies.

## Conclusions

Even minor compression from clothing can cause severe lower limb necrosis in frail elderly patients with edema. Early recognition and decompression, followed by stepwise drainage and wound management, can lead to full recovery without hospitalization. This case underscores the potential for effective, multidisciplinary wound care in the home medical environment.
